# Hypoxia and dehydroepiandrosterone in old age: a mouse survival study

**DOI:** 10.1186/1465-9921-7-144

**Published:** 2006-12-18

**Authors:** Edouard H Debonneuil, Janine Quillard, Etienne-Emile Baulieu

**Affiliations:** 1 Institut National de la Santé et de la Recherche Médicale, Unité Mixte de Recherche 788. Pincus Building, 80 rue du Général Leclerc, 94276 Le Kremlin-Bicêtre Cedex, France; 2 Service d'Anatomo-Pathologie, Hôpital de Bicêtre, Assistance Publique-Hôpitaux de Paris, Le Kremlin-Bicêtre, France

## Abstract

**Background:**

Survival remains an issue in pulmonary hypertension, a chronic disorder that often affects aged human adults. In young adult mice and rats, chronic 50% hypoxia (11% FIO2 or 0.5 atm) induces pulmonary hypertension without threatening life. In this framework, oral dehydroepiandrosterone was recently shown to prevent and reverse pulmonary hypertension in rats within a few weeks. To evaluate dehydroepiandrosterone therapy more globally, in the long term and in old age, we investigated whether hypoxia decreases lifespan and whether dehydroepiandrosterone improves survival under hypoxia.

**Methods:**

240 C57BL/6 mice were treated, from the age of 21 months until death, by normobaric hypoxia (11% FIO2) or normoxia, both with and without dehydroepiandrosterone sulfate (25 mg/kg in drinking water) (4 groups, N = 60). Survival, pulmonary artery and heart remodeling, weight and blood patterns were assessed.

**Results:**

In normoxia, control mice reached the median age of 27 months (median survival: 184 days). Hypoxia not only induced cardiopulmonary remodeling and polycythemia in old animals but also induced severe weight loss, trembling behavior and high mortality (p < 0.001, median survival: 38 days). Under hypoxia however, dehydroepiandrosterone not only significantly reduced cardiopulmonary remodeling but also remarkably extended survival (p < 0.01, median survival: 126 days). Weight loss and trembling behavior at least partially remained, and polycythemia completely, the latter possibly favorably participating in blood oxygenation. Interestingly, at the dose used, dehydroepiandrosterone sulfate was detrimental to long-term survival in normoxia (p < 0.05, median survival: 147 days).

**Conclusion:**

Dehydroepiandrosterone globally reduced what may be called an age-related frailty induced by hypoxic pulmonary hypertension. This interestingly recalls an inverse correlation found in the prospective PAQUID epidemiological study, between dehydroepiandrosterone blood levels and mortality in aged human smokers and former smokers.

## Background

In human beings, pulmonary hypertension (PH) is a chronic and life threatening disorder in which a progressive increase of pulmonary vascular resistance leads to right ventricular failure. When detected, PH is often an already irreversible chronic pathology and leads to death after several years of severe illness and treatment [1-5]. Among various etiologies, PH often develops in aged smokers with hypoxemia associated with chronic obstructive pulmonary disease (COPD) [6-10]: in these cases survival can be extended by long-term oxygenotherapy [9-13].

Therapies under development may be studied in rats and mice by their effects on pulmonary arterial pressure or cardiopulmonary remodeling. Survival has been studied in rats with the use of monocrotaline injection to model PH [14-17], but the multiple disorders caused [18-20] and the brief period over which deaths are recorded [14-17] bias long-term PH survival analysis. In fact, PH may not be deadly in itself: young adult mice and rats survive and develop stable PH within 3 weeks of 50% hypoxia (11% FIO2 or 0.5 atm) ([21-24], plus recurrent personal observation), and it was recently shown in rats that if hypoxia (0.5 atm) is maintained death does not occur until the rats are aged [25]. Since heart failure does occur in human PH, this brings into question today's development of PH therapies and their specific long-term global effects in laboratory animals.

Therefore we decided to use hypoxia, up to death in mice, starting at an age when they naturally start dying, in order to evaluate long-term positive or negative survival effects of hypoxic PH and a potential therapy. We considered dehydroepiandrosterone (DHEA), that has recently been shown to prevent and treat chronic hypoxic PH in rats when administered orally in its free (30 mg/kg every other day, 0.5 atm, [23]) or sulfate form (DHEAS; 9 mg/kg/day in drinking water, 11% FIO2, [24]; after oral ingestion most if not all the sulfate is converted into the free form).

Hypoxic pulmonary vasoconstriction helps oxygenating the blood but increases pulmonary arterial pressure. By relaxing contracted pulmonary arteries [23,26,27], DHEA inhibits both phenomena. Like any vasodilator it may therefore treat PH without being beneficial to the patient. Survival of aged mice will be our indicator of potential benefits. The old age is moreover of interest both because in humans PH complicating COPD often concerns aged persons [2-13] and because aged persons have lower blood DHEA(S) levels [28].

## Methods

### Conditions

Mice were obtained at the age of 17 months (240 C57BL/6 males from Elevage Janvier, Le Genest-St-Isle, France) and randomly distributed into 4 groups (N = 60) in cages containing 7 to 9 mice each with ad libitum standard diet (M20, Special Diet Services Ltd., Witham, Essex, UK) and water. At the age of 21 months – which we will refer to as t = 0 – each group received a different environmental condition, defined by a combination of hypoxia or normoxia and DHEA or not. Cages were changed weekly and food and drink renewed every other week. All procedures concerning animal care and use were carried out in accordance with the European Community Council Directive (86/609/EEC). All animal procedures were approved by the animal care and use committee at the institute. All treatments and measures were performed by investigators blinded to the treatment.

We chose normobaric hypoxia (11% FIO2) to avoid potential harmful consequences of rapid pressure variations. Hypoxic mice were housed in a home-made chamber homogeneously supplied by a flow of a filtered mixture of air and nitrogen (provided by a nitrogen generator from Air Liquide, Paris, France) at ambient pressure and 11 ± 1% oxygen (controlled by a ProOx controller from Biospherix, New York City, NY, USA). Control normoxic mice were housed in a similar chamber supplied by a flow of filtered air. Gas flowed sufficiently fast (15 l/min) into the chambers to ensure low carbonic gas levels (less than 0.05%). Hypoxia was interrupted weekly for roughly one hour for animal care.

DHEAS (Steraloids, Newport, RI, USA) was incorporated at 0.25 mg/ml (0.1 mg/ml gave partial results in rats, [24]) into the drinking water, except during the first two weeks where 0.1 mg/ml was used to allow taste habituation [29,30].

### Measurements

Survival was checked every one to three days until t = 180 days (when most animals had died in all groups). From time to time mice were weighed and their food and drink consumption was approximated by giving 350 g food and 500 ml drink per cage and measuring how much remained one week later.

Cardiopulmonary remodeling was measured in mice that died before t = 90 days (kept at -20°C when found – usually up to one day after death – up to analysis). Right ventricular hypertrophy was assessed by the right ventricle to left ventricle plus septum weight ratio (RV/LV+S) [23]. Lungs were formalin-fixed for histological study and pulmonary artery remodeling was expressed as percentage vessel wall thickness (100 × (external diameter-internal diameter)/external diameter, measured on a computer screen) in small and medium-sized pulmonary arteries (80–150 μm), averaged over 10 pulmonary arteries per mouse [23].

Blood sampling was performed on one initially randomly chosen cage per group. Additional cages were randomly chosen if needed to have at least 5 mice tested per group. The mice to be tested were placed in clean cages with their usual drink but no food overnight, and were excluded from survival analysis. Blood sampling (300 μl) was performed retro-orbitally under inhaled isoflurane anesthesia, in the morning. Blood was mixed with 10% ethylenediaminetetraacetic acid at 0.5 M. A blood analyzer (ABC-Animal Blood Counter, Scil, Viernheim, Germany) provided hematocrit, hemoglobin content, and the count, volume and hemoglobin concentration of red blood cells.

### Statistics

Values are expressed as mean ± SEM. Statistics were performed with JMP 6.0 (SAS Institute, Cary, NC, USA). Comparisons between two and several groups were done by Student and one-way ANOVA tests, respectively. Survival curve characteristics and comparisons were based on the proportional hazards Cox model. The method for choosing the number of animals is provided in an online additional file [see Additional file 1].

## Results

### Survival

Survival is clearly the main global health indicator. Note that mortality may affect the significance of results by death selection.

#### Before treatment: low mortality

It is rather unusual to start lifespan experiments with animals that are already aged. We wanted to start treatment (hypoxia or normoxia and DHEA or not) when the rate of 'natural death' becomes signifiant in C57BL/6 laboratory male mice. Starting with mice that are too old would imply that selection by death has comenced and that only resistant mice are being studied. If the mice are too young then in the short term no natural death will occur and any survival improvement due to a therapy may not be detected.

It appeared from the literature [31,32] that the appropriate starting age was 20 months. In fact our mice survived better than expected and we decided to start the treatments at the age of 21 months, with 5 deaths (plus 13 following arrival) instead of 20 or 25 as expected by extrapolating the literature. The results were then considered in terms of two 3-month time periods.

#### First 3 month period of treatment: dehydroepiandrosterone reduces a drastic age-specific hypoxic mortality

**Figure 1:**
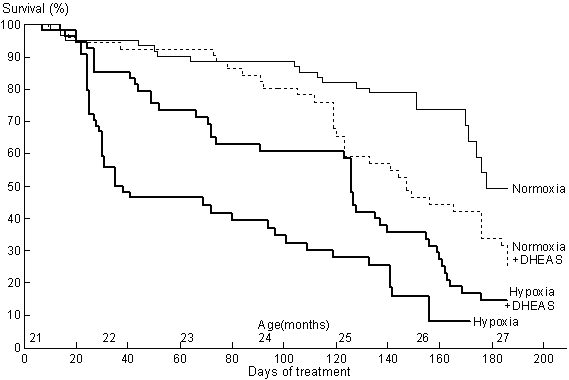
**Survival**. Survival of 21-month-old male C57BL/6 mice under hypoxia or normoxia (thick or thin lines), with or without dehydroepiandrosterone (dashed or solid lines). Hypoxia induced a high mortality. Dehydroepiandrosterone sulfate (DHEAS) prevented it, despite detrimental effects perceived in normoxia, at the oral sulfate dose used.

**Figure 2:**
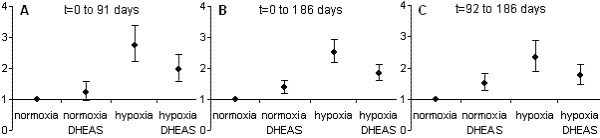
**Relative risk of death**. Relative risk of death taken from Figure 1, with normoxia as a reference and at time intervals: (A) t = 0 to 91 days (B) t = 0 to 186 days (C) t = 92 to 186 days. Despite temporary mortality patterns hypoxia and dehydroepiandrosterone (DHEAS) appear to globally have similar effects on survival at the three intervals.

Survival curves are shown in Figure 1 (t = 0 to 91 days; 21 to 24 month old mice) and relative risks of death for that period are shown in Figure 2a. Control mice – normoxia without DHEA – had a higher death rate than before the age of 21 months but there was still 89% survival at 24 months (compared to an expected ~70% from the literature). DHEA did not affect survival under normoxia (82% survival, relative risk of death: 1.24, p = 0.40). However, for hypoxic mice – without DHEA – the death rate increased drastically between t = 20 and t = 40 days, leading to only 48% survival, and then they died at a lower rate, leading to 39% survival at 24 months (relative risk of death: 2.73, p < 0.001 compared to control). Under hypoxia, DHEA led to 61% survival at 24 months with a roughly constant death rate: this treatment improved survival of hypoxic mice (relative risk of death: 0.68, p = 0.0065) while the normoxic survival level was not reached (relative risk of death: 1.62, p < 0.013).

#### Second 3 months of treatment: various age-related deaths

Over the next 3 months of treatment (24 to 27 month old mice, t = 92 to 183 days, figures 1 and 2c), mortality largely increased in all groups. Under normoxia, the control group reached 75% and 50% survival at 26 and 27 months (24 and 26 months would have been expected from the literature), and fewer died than in the 3 other groups (relative risk of death: 0.66, 0.43, and0.57; p = 0.014, p < 0.001 andp = 0.0014; compared to DHEA, hypoxia, and hypoxia+DHEA, respectively). The only statistical difference among the 3 groups was that normoxic mice with DHEA had a lower death rate than hypoxic mice without DHEA (p = 0.05).

#### In summary

Over the 6 months of treatment (21 to 27 month-old mice, t = 0 to 186 days, figure 1 and 2b) hypoxia induced a much higher mortality (median survival: 38 days, relative risk of death: 2.53, p < 0.001) than for control animals (mean survival: 184 days). DHEA globally improved survival under hypoxia (median survival: 126 days, relative risk of death: 0.72, p = 0.0025) but reduced it under normoxia (median survival: 126 days, relative risk of death: 1.39, p = 0.0025), compared with the corresponding untreated group.

### Cardiopulmonary remodeling

**Figure 3:**
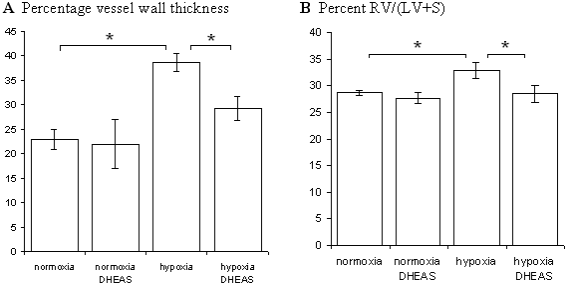
**Cardiopulmonary remodeling**. (A) Pulmonary artery remodeling (B) Heart remodeling in mice dead between t = 0 and 91 days. Hypoxia induced cardiopulmonary remodeling and dehydroepiandrosterone (named DHEAS in the figure) prevented it (*: p < 0.05).

**Figure 4:**
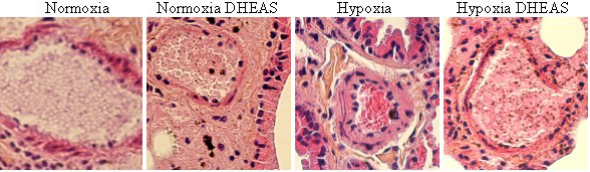
**Pulmonary artery sections**. Typical pictures of pulmonary arteries from mice under different conditions (image width: 150 μm). Hypoxic mice without dehydroepiandrosterone (DHEAS) have a thicker vessel wall with respect to diameter.

After death, PH can be diagnosed by the consequential increase in pulmonary artery wall thickness and enlarged right ventricule. We assessed cardiopulmonary remodeling in mice that died before t = 91 days (analysis of later deaths would lead to complex interpretations because of previous death selection and multiple age-related pathologies). Pulmonary artery remodeling (percentage vessel wall thickness) is shown in figure 3A (typical micrographs in figure 4) and heart remodeling (RV/LV+S percentage) in figure 3B.

Compared to the control group, hypoxic mice had higher pulmonary artery (38 vs 23; p = 0.01) and heart (0.325 versus 0.287; p = 0.05) remodeling. DHEA had no effect on the normoxic cardiopulmonary system but under hypoxia DHEA significantly reduced pulmonary artery and heart remodeling (29 vs 38; p < 0.05 and 0.286 versus 0.325; p < 0.05).

### Food and drink consumption

Overall, the mean daily consumption was of 3.0 ± 1 g and 3.25 ± 0.28 ml per mouse, with no particular distinction over groups and time. The consumption may have been lower because the determination did not take into account food and drink remaining at the bottom of the cage, which depend on the number of mice per cage, on their activity and on cage manipulation during the week. For DHEA-treated mice weighing ~30 g, we estimate that the DHEAS consumption was on the order of 25 mg/kg/day.

### Body weight

**Figure 5:**
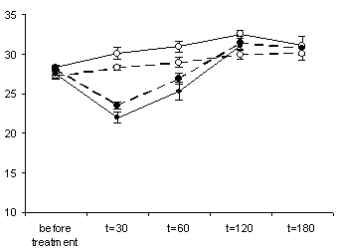
**Weight**. Weight of mice, two months before and after 30, 60, 120 and 180 days of treatment, under normoxia (empty circles) or hypoxia (filled circles), with (dotted line) or without (continuous line) dehydroepiandrosterone in their drinking water. Hypoxia induced a temporary weight loss, with and without dehydroepiandrosterone (*: p < 0.05) (it may be assumed that all the t = 0 points should coincide).

Sick mice generally lose weight and as such body weight (figure 5) may be used as an overall evaluation of the state of health.

#### Before treatment

When the mice arrived, we observed that they were thin (the mice had a similar diet before arrival, so the weight loss is probably due to the stress of transportation). The mice regained normal appearance within a month. When measured two months before treatment, all groups had similar weights (27.9 ± 0.12 g) and food and drink consumption.

#### Normoxic animals

Weights of control mice gradually increased until the age of 25 months (by 0.64 g/month, reaching 32.6 ± 0.1 g at t = 120 days, figure 5). This is probably a long increase towards a higher equilibrium weight long after the transportation weight loss (similar long-term weight changes are observed after changing diets [32]). The weight then slightly (but not significantly) decreased on average (figure 5), which may reflect negative selection of heavy animals by death. DHEA-treated normoxic mice also gained weight but to a lower extent (by 0.42 g/month up to t = 120 days), weighing slightly but significantly less (p ~ 0.007) than control mice at t = 30, 60 and 120 days.

#### Hypoxic animals: temporary weight loss and trembling behavior

After two weeks of hypoxia, all aged mice, with and without DHEA, were particularly thin and for many, if not all of them, normal cage behavior was interrupted by periods of trembling while curling up. When measured after one month of treatment, the weight of hypoxic mice was indeed much lower than their normoxic counterparts (22 ± 0.7 g versus 30.1 ± 0.8 g, p < 0.001). After two or three months, the -remaining- mice regained normal size (and normal weight, figure 5) and trembling behavior became rare. The trembling behavior also occured with DHEA. For weight, DHEA did not obviously reduce the hypoxic weight loss (23.6 ± 0.5 g versus 22 ± 0.7 g, p = 0.11 at t = 30 days), but an already large selection by death in the hypoxic group without DHEA could mask the difference.

### Hematocrit

**Figure 6:**
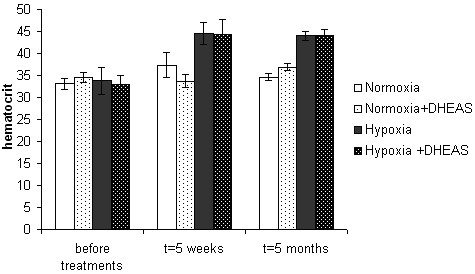
**Hematocrit**. Hematocrit as a function of groups and time. Hypoxia increased the hematocrit, and dehydroepiandrosterone (DHEAS) did not affect the hematocrit, in hypoxia or in normoxia.

The evolution of the hematocrit among groups is shown in figure 6 and other blood parameters in table 1. Hypoxia typically induces polycythemia which may compensate for the lack of oxygen [33] and is caracterized by a high hematocrit.

**Table 1:** Blood patterns

	Treatment		before treatments	t = 5 weeks	t = 5 months	
Blood hemoglobin content (g/dl)	Normoxia	Water	10.6 ± 0.5 (6)	12.4 ± 0.3 (7)	11.3 ± 0.9 (7)	**
	Normoxia	DHEAS	11.3 ± 0.3 (9)	11.5 ± 0.4 (7)	12.0 ± 0.2 (5)	
	Hypoxia	Water	11.0 ± 0.4 (6)	14.3 ± 0.3 (6)	13.9 ± 0.6 (8)	
	Hypoxia	DHEAS	11.3 ± 0.7 (6)	14.3 ± 0.9 (6)	14.3 ± 1.2 (8)	

Hematocrit (%)	Normoxia	Water	7.86 ± 0.3 (6)	8.65 ± 0.3 (7)	7.97 ± 0.7 (7)	**
	Normoxia	DHEAS	8.03 ± 0.4 (9)	7.83 ± 0.3 (7)	8.61 ± 0.2 (5)	
	Hypoxia	Water	7.81 ± 0.3 (6)	9.70 ± 0.3 (6)	9.29 ± 0.5 (8)	
	Hypoxia	DHEAS	7.74 ± 0.6 (6)	9.68 ± 0.5 (6)	9.21 ± 0.7 (8)	

Red cell count (10^3^/mm^3^)	Normoxia	Water	42.2 ± 0.3 (6)	43.1 ± 0.5 (7)	42.9 ± 0.6 (7)	**
	Normoxia	DHEAS	43.1 ± 0.5 (9)	43.2 ± 0.7 (7)	43.0 ± 0.6 (5)	
	Hypoxia	Water	43.3 ± 0.4 (6)	46.0 ± 0.8 (6)	47.5 ± 0.4 (8)	
	Hypoxia	DHEAS	42.9 ± 0.4 (6)	46.2 ± 1.8 (6)	47.8 ± 1.2 (9)	

Mean red blood cell volume (μm3)	Normoxia	Water	42.2 ± 0.3 (6)	43.1 ± 0.5 (7)	42.9 ± 0.6 (7)	*
	Normoxia	DHEAS	43.1 ± 0.5 (9)	43.2 ± 0.7 (7)	43.0 ± 0.6 (5)	
	Hypoxia	Water	43.3 ± 0.4 (6)	46.0 ± 0.8 (6)	47.5 ± 0.4 (8)	
	Hypoxia	DHEAS	42.9 ± 0.4 (6)	46.2 ± 1.8 (6)	47.8 ± 1.2 (9)	

Mean cell hemoglobin concentration (g/dl)	Normoxia	Water	32.1 ± 0.2 (6)	33.1 ± 0.2 (7)	32.8 ± 0.3 (7)	
	Normoxia	DHEAS	32.6 ± 1.4 (9)	34.1 ± 0.3 (7)	32.6 ± 0.3 (5)	
	Hypoxia	Water	32.8 ± 0.4 (6)	32.0 ± 0.5 (6)	31.5 ± 0.3 (8)	
	Hypoxia	DHEAS	34.6 ± 0.3 (6)	32.4 ± 0.2 (6)	32.4 ± 0.3 (8)	

Mean cell hemoglobin (pg)	Normoxia	Water	13.5 ± 0.1 (6)	14.3 ± 0.2 (7)	14.1 ± 0.3 (7)	
	Normoxia	DHEAS	14.1 ± 0.7 (9)	14.7 ± 0.1 (7)	14.0 ± 0.2 (5)	
	Hypoxia	Water	14.2 ± 0.2 (6)	14.7 ± 0.1 (7)	15 ± 0.1 (8)	
	Hypoxia	DHEAS	14.9 ± 0.2 (6)	14.9 ± 0.5 (6)	15.4 ± 0.3 (8)	

Red blood parameters for the different treatments at different times. Blood hemoglobin content, hematocrit and red cell count were elevated under hypoxia compared to normoxia, at t = 5 weeks (p < 0.01) and similarly at t = 5 months (p < 0.01) (**). Mean red blood cell volume was slightly elevated under hypoxia compared to normoxia, at t = 5 weeks (p < 0.05) and slightly more at t = 5 months (p < 0.05) (*). Mice treated with dehydroepiandrosterone (named DHEAS in the table) had the same blood patterns than matching mice that did not received dehydroepiandrosterone (named Water in the table), whether under normoxia or hypoxia.

One month before treatment, all groups had a similar hematocrit (figure 6). Under normoxia the hematocrit remained the same, at t = 5 weeks (t = 33 to 37 days) as well as at t = 5 months (~150 days), with or without DHEA.

As expected, hypoxia increased the hematocrit. The hematocrit reached similar levels (45%) at t = 5 weeks and t = 5 months. The same trend was observed for red blood cell counts and blood hemoglobin content, while cellular hemoglobin content remained unchanged.

DHEAS did not affect the hematocrit nor red blood cell properties, neither in normoxia nor in hypoxia, at t = 5 weeks and t = 5 months.

## Discussion

1. Hypoxia induced PH in old mice and DHEA prevented it. 2. Hypoxia drastically induced mortality and weight loss in old age. 3. In its sulfate form and at the used oral dose DHEA was detrimental to long-term survival in normoxia. 4. DHEA however largely prevented hypoxic death during the whole experiment.

### DHEA prevents hypoxic PH in old mice

#### Chronic hypoxia provoked PH in old mice

This is not particularly surprising as it also does it in young adult mice [21] and rats [23,24].

#### DHEA prevented hypoxic PH in mice

DHEA has already been shown to prevent and reverse PH in rats [23,24]. DHEA is thought to be the relaxation of pulmonary arteries by opening large-conductance calcium-activated potassium channels [23,26,34], but this mechanism is controversial [26,27]. Mice knocked out for these channels [35-37] exist and it would be of interest to study the relaxation of pulmonary arteries by DHEA in such mice.

#### DHEA prevented hypoxic PH in old age

No previous study reported effects of DHEA on *PH *in old age. Old age is a common factor for *PH *incidence and low endogenous blood DHEA(S) levels in humans [28]. Therefore old age may play a particular role in the treatment of hypoxic PH by DHEA, and it was not obvious that results obtained in young adults could be transposed to old adults (especially from rats to mice). Application to humans is discussed further along with survival.

### Hypoxic death in old animals: a model for PH survival?

*We used old animals *at an age when they naturally die in order to measure overall positive or negative health effects by increased or decreased survival of 'naturally dying' animals. Our mice trembled and there was a drastic increase of death due to hypoxia (11% FIO2). To our knowledge this has not been described before and it is certainly due to the old age of the mice. In particular we also studied young adult mice (8 with DHEA and 16 without, unpublished data) for 4 months in the same hypoxic chamber, with no trembling behavior nor death (p < 0.001).

*This age-related frailty to chronic hypoxia *was not foreseen. In particular, there does not seem to be an age-related frailty with respect to severe acute hypoxia [38,39]. In other species, flies and nematodes live longer under moderate hypoxia, possibly because of reduced oxidative stress, and it could be expected that the same might apply to mammals [40]. Our degree of hypoxia (11% oxygen) was clearly too severe to allow mice to benefit from reduced oxygen stress but a less severe degree (16% oxygen, unpublished data) still slightly reduced lifespan. Starting hypoxia at a younger age still reduces lifespan: a recent study has shown that rats kept under hypoxia from a young adult age rapidly develop cardiopulmonary remodeling and die when they are around 18 months old [25]. These rats were Wistar rats, which have a similar lifespan to C57BL/6 mice. If we suppose that hypoxia has similar effects on survival in both strains, this suggests that hypoxia only threatens life after ~18 months of age, whatever the duration of hypoxia before that age. The combination of this rat study with our mouse study suggests that in mammals, although hypoxic PH develops within a few weeks at any age, hypoxic PH becomes dangerous for health at later ages rather than after some disorder duration.

*In humans too*, there could be an age-related frailty to PH. It happens that the incidence of hospitalization and mortality from the disorder increases exponentially with age [5]. Moreover, there seems to be an age, around 45 years, when pulmonary arterial hypertension becomes life-threatening [4]. In fact hypoxic PH severity could be more related to patient age than disease duration. This could perhaps explain why apparently minor PH may be determinant for (older) COPD patients [10], and why some old smokers suddenly suffer after many years of COPD. Of course, this age-related concept does not concern all types of PH (such as PH in the newborn and probably fenfluramine-induced PH, [3]).

*We propose that our model *– consisting of studying survival of old animals under hypoxia accompanied or not by some treatment – may be useful for studying the overall effects of PH treatments which are destined for aged persons. If we accept the difference that time goes 30 to 40 times faster in mice, there is a surprisingly good match between our survival curves of old hypoxic mice, treated or not by DHEA, and the survival curves of COPD patients, mostly over 65 years old, treated or not by oxygenotherapy [13]. This, may suggest that hypoxic mice survival could be a speeded-up model for human PH survival.

### DHEA was detrimental to long-term survival

*An appropriate control *should not affect survival and should be transposable to humans. However DHEA induced an unexpected decrease of survival after the age of 24 months compared to the control mice (p = 0.0025), and this may not at all apply to humans. In humans it was shown that DHEA may be safely administered to older persons at the daily oral dose of 50 mg (~1 mg/kg/day) for one year [28]. In comparison, the doses used to treat PH in animals are larger (~9 mg/kg/day by Hampl V *et al*. [24], ~15 mg/kg/day by Bonnet *et al*. [23] and ~25 mg/kg/day in our study). In fact, whereas in humans DHEA(S) is a major steroid circulating in the blood, no detectable DHEA(S) was found in the blood of laboratory animals such as mice or rats [41]. Therefore, DHEA "supplementation" is pharmacological (i.e. non physiological) in mice and cannot be considered as a hormonal replacement therapy.

*The effect on lifespan of DHEA administration in mice *has been studied several times. High doses of free DHEA incorporated into the diet (on the order of 0.4%, which corresponds to ~12 mg/day/mouse, that is 10 to 20 times more than in our study) have been shown to increase the lifespan of particular short-lived mice [42-44]. As C57BL/6 mice do not seem to like DHEA [29,30,45], we prefered to use lower doses and the sulfate form in drinking water (0.25 mg/ml dissolves well in water) to avoid survival bias by caloric restriction, and we found that it reduced the lifespan of 21-month-old male C57BL/6 mice.

*We are not the first to find that DHEAS *does not extend the lifespan of mice. A previous study found that 10 times less DHEAS (0.025 mg/ml in drinking water) did not affect the lifespan of 12-month-old male C57BL/6 mice [31]. The authors suggested that the lack of effect could come from an insufficient dosage. Another study found that the intermediate dose of 0.1 mg/ml in drinking water from weaning age insignificantly decreased the lifespan of genetically heterogeneous mice [46]. We multiplied the dose by 3 and the decrease of lifespan became very significant. Although multiple parameters make the comparisons complex, a global interpretation of these results would be that DHEAS in drinking water does not affect mouse lifespan at doses smaller than 0.1 mg/ml (~9 mg/kg/day) and decreases mouse lifespan at larger doses. In fact, positive effects of dehydroepiandrosterone may be present but masked by negative effects due to the dose and way of administration, such as long-term hepatic disturbances [47][48].

### DHEA largely prevented hypoxic death

#### DHEA globally treated hypoxic old mice

Although DHEAS administration appeared to be detrimental in the long term (as seen by late mortality under normoxia), and although hypoxic animals treated by DHEA still lost weight and trembled, DHEA largely (but not completely) prevented the hypoxic mortality over the whole experiment. This overall beneficial survival effect is the best possible answer to our questions: DHEA not only treats hypoxic PH but also hypoxic (old) mice.

#### A role for high hematocrit?

The vasorelaxation of pulmonary arteries by DHEA could have led to overall negative effects since hypoxic vasoconstriction of pulmonary arteries is useful to improve blood oxygenation. The question arises of whether, with DHEA treatment, the body managed without the oxygen provided by vasoconstriction or another mechanism for providing an adequate oxygen supply came into play. The high blood hemoglobin content here may play a role. By preventing cardiopulmonary remodeling but permitting increased hematocrit under hypoxia, DHEA could be favorable to the animal's health by preventing heart failure (due to PH) while allowing high oxygenation.

*The prevention of hypoxic death by DHEA in mice recalls us the prospective PAQUID study in humans*, where a strong inverse correlation between natural DHEA(S) blood levels and the ten year mortality in old male smokers and former smokers has been reported [49]. There is an interesting analogy between ≥ 65-year-old male human smokers and ≥ 21-month-old male hypoxic mice, on the time scale of the mouse. This analogy is important as we designed our mice survival study with the results of the PAQUID study in mind. Nevertheless it must be remembered that mice, unlike humans, do not have detectable endogenous circulating DHEA(S) [41]. Therefore the above analogies compare pharmacological (mice) effects with physiological/pharmacological (human) effects. It remains that large doses of DHEA may be safely administered to humans and that PH complicating COPD is a morbid condition. Thus it seems that specific human clinical trials aimed at deriving statistics from humans taking DHEA supplementation, and including females who have not been taken into account in this (mouse) study, would be justified. In the meanwhile, care should be taken to avoid uncontrolled consequences of our findings.

## Conclusion

There seems to be a frailty to hypoxic PH that is particular to old age, in mice and possibly in humans. This suggests that survival studies with aged mice under hypoxia may be pertinent for evaluating therapies for aged patients having PH. In that framework, DHEA was found to remarkably improve survival under hypoxia. The comparison between mice and humans is not obvious, but our findings interestingly resemble human observations, that together suggest trials of DHEA treatment to PH and COPD in humans.

## Abbreviations

FIO2: Fraction of Inspired Oxygen

PH: Pulmonary Hypertension

COPD: Chronic Obstructive Pulmonary Disease

DHEA(S): DeHydroEpiAndrosterone (sulfate)

## Competing interests

This work was financed by the Association pour la Recherche sur les Nicotianés (Fleury-Les-Aubrais, France).

## Authors' contributions

EHD carried out the design of the study, performed the statistical analysis, carried out the environmental setting, participated in blood analysis, anatomopathological analysis and drafted the manuscript. JQ carried out the anatomopathological analysis and helped to design the study. EEB participated in design and coordination of the study and helped to draft the manuscript.

## Supplementary Material

Additional file 1 SurvivalPower Click here for file
