# Supplementary material for: Hypoxia and dehydroepiandrosterone in old age: a mouse survival study
Source: Respir Res. 2006 Dec 18;7(1):144. doi: 10.1186/1465-9921-7-144 (PMC1764736; doi:10.1186/1465-9921-7-144)
Supplement: Additional file 1 — SurvivalPower [file 1465-9921-7-144-S1.pdf]

ADDITIONNAL FILE – SurvivalPower.doc

Statistics to determine the number of animals to be studied.

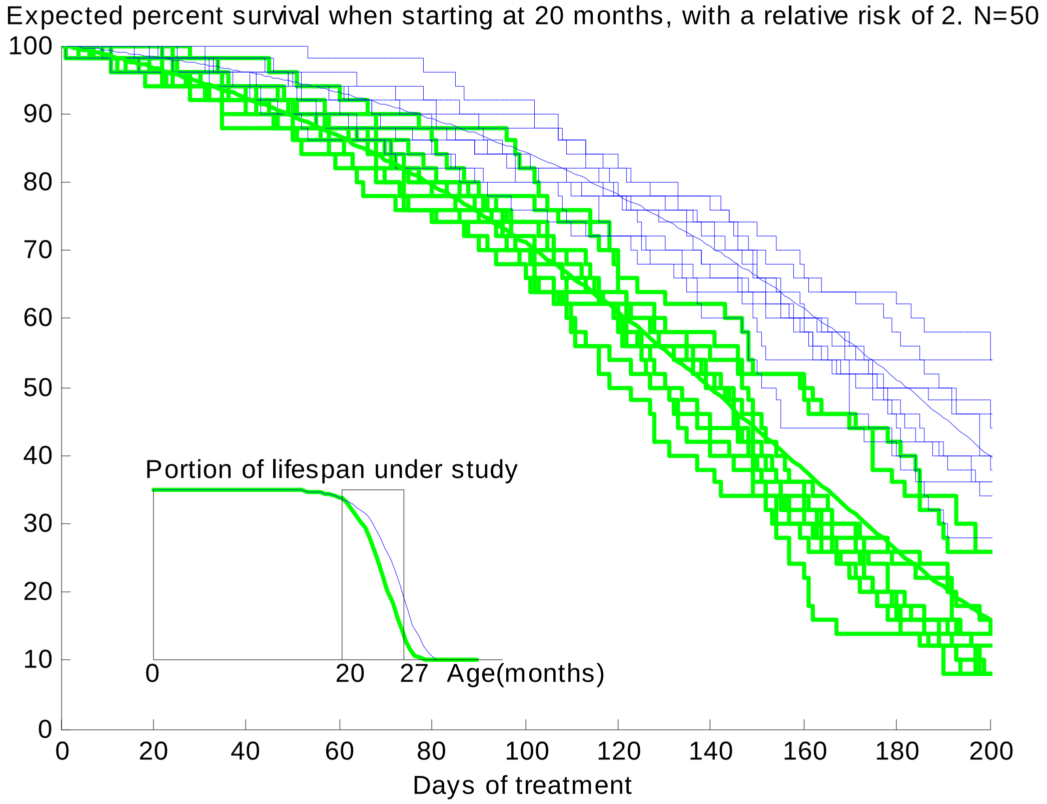

To determine the number of animals to be studied, computer simulations of normal lifespans (10 blue curves here) and accelerated lifespans (10 green thick curves; relative risk 2 at dying) were made for the portion of lifespan being studied (from 20 to 26 months). The more animals used, the closer lifespans are to their theoretical distribution (continuous curves). Here we used the Gompertz theoretical distribution, defined by an underlying exponentially increasing risk of dying. We adjusted the parameters to literature [30,31].

Visualization of any two curves in most cases allow estimation of whether they belong to the same group (same color) or not. The log rank statistical test was used for precision. If two curves are of different color the power of discrimination is 80% at $p<0.05$, if they are of the same color the risk (alpha) of discrimination is 5% at $p<0.05$.

These curves were obtained with N=50. Extending the time of observation only slightly improves detection because few animals remain. Detection of smaller differences would require more animals but this was not considered necessary. We used N=60 to account for additional death before the beginning of treatment and for additional mice for blood sampling.
